# Complete mitochondrial genome of the Korean flying fish *Cheilopogon doederleinii* (Beloniformes, Exocoetidae): mitogenome characterization and phylogenetic analysis

**DOI:** 10.1080/23802359.2016.1258346

**Published:** 2016-11-22

**Authors:** Seungki Lee

**Affiliations:** Biological and Genetic Resources Assessment Division, National Institute of Biological Resources, Incheon, Republic of Korea

**Keywords:** Mitochondrial genome, Beloniformes, Exocoetidae, *Cheilopogon doederleinii*

## Abstract

The Korean flying fish, *Cheilopogon doederleinii*, is a marine fish species belonging to the family Exocoetidae. In this study, I report for the first time the sequencing and assembly of the complete mitochondrial genome of *C. doederleinii*. The complete mitochondrial genome is 16,525 bp long and includes 13 protein-coding, 22 tRNA, and 2 rRNA genes. It has the typical vertebrate mitochondrial gene arrangement. Phylogenetic analysis using mitochondrial genomes of 10 species showed that *C. doederleinii* is clustered with *C. arcticeps* and grouped with the other Exocoetidae species. This mitochondrial genome provides potentially important resources for addressing taxonomic issues and studying molecular evolution.

The Korean flying fish, *Cheilopogon doederleinii*, is a marine fish species belonging to the family Exocoetidae. This species is widely distributed in the western Pacific Ocean and is commercially fished in Korea, Japan, and China (Kim et al. 2005). To date, there has been very little molecular and genetic research on this species. To the best of my knowledge, this is the first study to determine the complete mitochondrial genome of *C. doederleinii* and to analyze the phylogenetic relationship of this species among Beloniformes fishes.

The *C. doederleinii* specimen collected from the Jeju Island in Korea (33.13N, 126.14E) was deposited in the National Marine Biodiversity Institute of Korea (Specimen No. MABIK PI00045941). The germeline stem cells of this species were cryopreserved in the National Institute of Biological Resources (Incheon, Korea) with a slight modification to previously reported methods (Lee et al. 2013; Lee et al. 2016). The mitochondrial genome was amplified in its entirety using a long PCR technique (Cheng et al. 1994). I used fish-versatile PCR primers in various combinations to amplify contiguous, overlapping segments of the entire mitogenome. All the experiments were carried out following previously described methods (Miya & Nishida 1999).

The complete mitochondrial genome of *C. doederleinii* (GenBank accession no. AP017897) is 16,525 bp long and includes 13 protein-coding, 22 tRNA, and 2 rRNA genes.

The *ND6* gene and eight tRNA genes are encoded on the light-strand. The overall base composition of the heavy-strand is 29.22% of A, 27.15% of C, 16.29% of G, and 27.31% of T. As in mitogenomes of other vertebrates (Saccone et al. 1999), the AT content is higher than the GC content. All tRNA genes can fold into a typical cloverleaf structure and are 65–74 bp long. The 12S rRNA (944 bp) and 16S rRNA genes (1686 bp) are located between *tRNA^Phe^* and *tRNA^Val^* and between *rRNA^Val^* and *tRNA^Leu(UUR)^,* respectively. Of the 13 protein-coding genes, 12 start with ATG; the exception being *COI*, which starts with GTG. Seven of the 13 protein-coding genes terminate with incomplete stop codons, T–– (*ND2*, *COII*, *ND3*, *ND4*, and *Cytb*) and TA– (*ATP6* and *COIII*), whereas the remaining six genes ended with complete stop codons (TAG and TAA). A control region (866 bp) is located between *tRNA^Pro^* and *tRNA^Phe^*.

Phylogenetic trees were constructed (maximum likelihood) with 1000 replicates using MEGA 7.0 software (MEGA, PA) (Kumar et al. 2016) for the newly sequenced mitogenome and a further nine complete mitogenome sequences downloaded from the National Center for Biotechnology Information. I confirmed that *C. doederleinii* is clustered with *C. arcticeps* (Chou et al. 2016) and grouped with the other Exocoetidae species (Figure 1). This mitochondrial genome provides potentially important resources for addressing taxonomic issues and studying molecular evolution.

**Figure 1.**
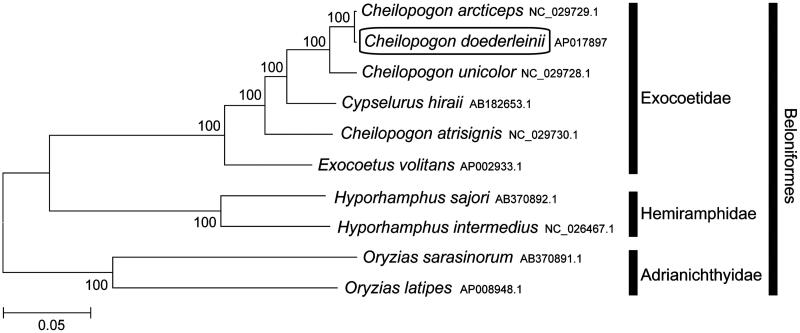
Phylogenetic position of *C. doederleinii* based on a comparison with the complete mitochondrial genome sequences of Beloniformes species. The analysis was performed using the MEGA 7.0 software. The accession numbers for each species are indicated after the scientific names.
